# Computer Vision-Based Path Planning for Robot Arms in Three-Dimensional Workspaces Using Q-Learning and Neural Networks

**DOI:** 10.3390/s22051697

**Published:** 2022-02-22

**Authors:** Ali Abdi, Mohammad Hassan Ranjbar, Ju Hong Park

**Affiliations:** 1 Department of Convergence IT Engineering, Pohang University of Science and Technology (POSTECH), Pohang 37673, Korea; abdiali@postech.ac.kr; 2 School of Mechanical Engineering, College of Engineering, University of Tehran, Tehran 11155-4563, Iran; mhranjbar@ut.ac.ir

**Keywords:** path planning, Q-learning, neural network, YOLO algorithm, computer vision, robot arm, target reaching, obstacle avoidance

## Abstract

Computer vision-based path planning can play a crucial role in numerous technologically driven smart applications. Although various path planning methods have been proposed, limitations, such as unreliable three-dimensional (3D) localization of objects in a workspace, time-consuming computational processes, and limited two-dimensional workspaces, remain. Studies to address these problems have achieved some success, but many of these problems persist. Therefore, in this study, which is an extension of our previous paper, a novel path planning approach that combined computer vision, Q-learning, and neural networks was developed to overcome these limitations. The proposed computer vision-neural network algorithm was fed by two images from two views to obtain accurate spatial coordinates of objects in real time. Next, Q-learning was used to determine a sequence of simple actions: up, down, left, right, backward, and forward, from the start point to the target point in a 3D workspace. Finally, a trained neural network was used to determine a sequence of joint angles according to the identified actions. Simulation and experimental test results revealed that the proposed combination of 3D object detection, an agent-environment interaction in the Q-learning phase, and simple joint angle computation by trained neural networks considerably alleviated the limitations of previous studies.

## 1. Introduction

Intelligent robot arms can play a crucial role in automation. The extensive use of industrial [1], surgical [2], and home robots [3] are examples of applications in which robot arms have become indispensable. Many robot arms are synchronized to accomplish a task by using a program or are remotely controlled by human operators. Intelligent robot arms attached with numerous sensors and cameras have attracted considerable research attention [4]. These robots have powerful onboard processors, high memory capacity, and artificial intelligence (AI)-based algorithms. These features enable such robots to replicate human capabilities. Intelligent robot arms gather information regarding their environment to make decisions in real time.

Intelligent robot arms are also increasingly being used in numerous applications. For instance, in smart cities [5], these kinds of robots are used to scan buildings and generate automated three-dimensional (3D) reconstruction. In computer vision and computer graphics, 3D reconstruction is used to detail the shape and appearance of physical objects, define 3D profiles, and determine the 3D coordinates of any point on the profile. Furthermore, 3D reconstruction has applications in many fields, such as medicine, free-viewpoint video reconstruction, robotic mapping, city planning, gaming, virtual environments, virtual tourism, landslide inventory mapping, robot navigation, archaeology, augmented reality, reverse engineering, motion capture, gesture recognition, and hand tracking [6].

In addition to 3D reconstruction, intelligent robot arms can play an essential role in other applications. For example, in smart factories [7], intelligent robot arms can be used in production, manufacturing, assembly, and packing in various industries. Moreover, in smart hospitals, robot-assisted surgery allows doctors to perform many types of complex procedures with higher precision, flexibility, and control than is possible with conventional techniques. In smart homes, robot arms can assist people with disabilities, elderly individuals, and parents rearing children to considerably improve their quality of life. The accuracy, speed, and efficiency of robot arms enable them to perform daily work effortlessly.

Although intelligent robot arms can be used in many industries, developing robotic arm applications remains highly challenging. One crucial and difficult problem is to ensure that robot arm path planning is accurate, safe, and efficient [8]. Path planning refers to bringing the end-effector to the target without hitting obstacles. It depends on various algorithms that dictate the motion of the robot arm, and determine how a robot arm should approach, process, and orient itself for optimal productivity and collision avoidance. Numerous computer vision-based path planning approaches were reviewed. Given that the end-effector of robot arms is critical for achieving their goal, bringing the end-effector to the desired location with obstacle avoidance is a key challenge for these robots.

This study aimed to overcome certain limitations of current computer vision-based path planning of robot arms by using AI techniques to develop an intelligent robot arm with high performance, safety, and speed.

To this end, although we tried to address those limitations in our previous paper [9], some of them remain. This article that is, in fact, an extension of our previous study, tries to address the limitations of prior work. In the previous study, we developed a novel hybrid path planning method using Q-learning and neural networks. An action finding (active approach) and angle finding (passive approach) were the two components of the hybrid path planning system. The Q-learning algorithm was used in the active phase to determine a series of simple movements, such as going up, down, left, and right to reach a target cell in a two-dimensional (2D) grid workspace. In the passive phase, a neural network is trained to determine the joint angles of the robot arm with respect to the observed actions. According to our findings, this hybrid technique considerably improved the speed and reduced the complexity of the operation of the system. However, this study had the following limitations.

1. Although many applications require 3D movement, the scope of work was limited to the 2D workspace.
2. Finding a start, an obstacle, and a target point through their colors may negatively affect image processing accuracy depending on the ambient light. Obstacle detection based on shapes would be preferable.
3. Because the KNN algorithm was used in this study, a start, an obstacle, and a target cell were required to have distinct colors.
4. Only one obstacle could be located in the workspace unless distinct colors were used for each obstacle.

Therefore, in this study, we extended the scope of our previously proposed method from a 2D space to a 3D space and incorporated real-time object detection and localization for real-world applications.

In this article, the proposed path planning method comprises three phases: (1) detecting the spatial coordinates of a start, target, and obstacle object, (2) finding the optimal path from a start to a target object while avoiding an obstacle object, and (3) calculating the corresponding angles for the six joints of a robot arm. In the first stage, the spatial coordinates of a start, target, and obstacle object were recognized in a 3D workspace using a combined object detection technique and a neural network with two images captured using two cameras from different views. This stage required a training process based on the created dataset of the start, target, and obstacle shapes and their morphologies. In the next step, the Q-learning algorithm is used to determine the optimal actions in a gridded 3D workspace so that a robot arm could begin traveling from the start cell to the target cell without collision with an obstacle. In a Q-learning algorithm, states are represented by cells in a 3D workspace, and forward, backward, right, left, down, and up are defined as actions of the robot arms. Finally, in the next step, a trained neural network is used to calculate the angles for the six joints of a robot arm based on actions to place the end-effector in the required location in 3D space. The use of a neural network for this stage greatly reduces the calculation time and computing cost.

This approach exhibited high speed, low computational cost, and automated path generation for various situations. A real-time object detection algorithm is obtained by combining neural network and object detection. Furthermore, finding optimal actions by Q-learning algorithm and calculating robot arm joint angles with trained neural networks enabled us to create a precise, efficient, and fast method to address computer vision-based path planning issues for real-life applications. The remainder of this paper is organized as follows. In Section 2, the related works are presented. In Section 3, our novel computer vision-based path planning technique is proposed. In Section 4, the experimental results are presented and analyzed. In Section 5, the discussion is presented. Finally, in Section 6, the conclusion is presented.

## 2. Related Works

Prior to the development of a new computer vision-based path planning approach, numerous existing approaches were reviewed. Methods, such as probabilistic road map (PRM), artificial potential field (APF), rapidly exploring random tree (RRT), and reinforcement learning (RL)-based approaches, have been proposed. The most important of which are as follows.

Ka et al. presented a vision-based assistive robot arm assistance algorithm for a JACO robot in which a low-cost 3D depth-sensing camera and an improved inverse kinematic algorithm were used to enable semiautonomous or autonomous JACO operation [10]. Rai et al. proposed an autonomous robotic framework for academic, vocational, and training purposes. They used two webcams that provided the top and side views to consider the objects of various heights for positioning a robotic gripper at the center of the target [11]. Hsu et al. proposed a control design and implementation of an intelligent vehicle combined with a robotic manipulator and computer vision [12]. Chen et al. demonstrated the potential of combining augmented reality-based brain–computer interface and computer vision to control robotic arms. They employed hue, saturation, and lightness space (HSV) to provide the object’s position and color in the 2D workspace. The objects they utilized were simply 2D disks with placements in a conventional gridding pattern [13]. Whang et al. used a popular object detection model, the faster R-CNN model, to detect nails and screws in construction waste recycling robots. Their result shows that the model’s mean average precision (AP) for nails and screws was 0.891. This precision was well in their application [14]. Tebbe et al. demonstrated an innovative table tennis robot system with high precision vision detection and rapid robot response. They used a multi-camera calibration approach and iterative triangulation to reconstruct the 3D ball position with a 2.0 mm precision. They used classic image processing techniques and integrated color and background thresholding to detect the flying ball with higher velocities in real-time [15]. Sadhu et al. proposed an improvised FA that involved the Q-learning framework within itself for robot arms path planning. In this proposed Q-learning induced FA (QFA), the optimal parameter values for each firefly of a population were learned by the Q-learning strategy during the learning phase and applied thereafter during execution [16]. Wen et al. presented a new obstacle avoidance algorithm based on deep deterministic policy gradient (DDPG). Specifically, they proposed to use DDPG to plan the trajectory of a robot arm to realize obstacle avoidance [17]. Zhang et al. proposed a path planning method based on Q-learning for robot arm due to its simple and well-developed theory [18]. Huadong et al. analyzed the characteristics of obstacle avoidance path planning to improve the efficiency and accuracy of obstacle avoidance path planning [19]. Das et al. proposed a novel method of energy-efficient path planning of an industrial robot arm in a workspace with multiple obstacles using differential evolution (DE) algorithm [20]. Raheem et al. analyzed the shortest path and trajectory planning of a two-link robot arm with 2-DOF in the 2-D static known environment [21]. Chang et al. presented an automatic path planning of a six-axis robot for intelligent manufacturing based on network remote controlling and simulation [22]. Sugiura et al. determined an optimal path by computing the gradient of an equation using the APF method [23]. However, the algorithm may encounter local minima, rather than the absolute minimum, and therefore the shortest path may not be identified, as reported by Martínez et al. [24]. Kavraki et al. used Dijkstra’s algorithm to calculate the shortest paths between nodes on a graph in the PRM method [25]. The path created by the sampling-based approach may not be optimal because the resultant path mainly depends on sample procedures, as reported by Hsu et al. [26]. Liu et al. reported that RRT can avoid precise environmental modeling and reduce calculations [27]. However, the convergence speed of motion planning is sluggish because of the random sampling and global uniform sampling technique of rapidly expanding random trees in redundant spaces. Therefore, producing the optimal path in a short period of time becomes challenging, as reported by Karaman et al. [28]. Prianto et al. used the soft actor-critic (SAC) deep-learning-based method for path planning. Because of the use of the entropy term in the goal function, the SAC exhibits high exploration capabilities for path planning [29]. Panov et al. investigated novel grid path planning with deep RL outcomes. Furthermore, they demonstrated the robust learning ability of a neural Q-learning agent on tiny maps and achieved promising results on new maps [30]. Low et al. suggested improved Q-learning and demonstrated its efficacy through experimental investigations [31]. Yu et al. proposed a neural-network-based path planning model for mobile robots based on hierarchical RL and compared this model to other algorithms. The results revealed the smoothness of the planned path and usable generalization in various scenarios by using an obstacle avoidance method based on non-uniform rational B-splines (NURBS) for robot arms [32]. It was also found that the NURBS method was highly effective for avoiding collisions [33].

These methods have distinct advantages and disadvantages. Certain methods have irregular paths, require preplanning, determine a non-optimal path, obtain cubic graphs, and are slow. Other methods have drawbacks, such as high complexity or are limited to certain conditions. Because robot arm performance cannot be generalized, some robot arm setups can become highly complex. The development of a novel path planning method that identifies and solves these limitations is essential for improving robot arm performance and production. The proposed approach meets this requirement with some advantages that will be presented in the next sections.

## 3. Methods

The proposed procedure can be summarized as follows: (1) detecting the coordinates of the bounding box of a start, target, and an obstacle by using an object detection algorithm. Then, converting them to their spatial coordinates using a neural network, (2) determining optimal actions using Q-learning, and (3) calculating the rotation angles of the joints of a six-degree-of-freedom robot arm using a neural network. This section describes these in detail.

### 3.1. Object Detection and Spatial Coordinates (Combined YOLO-Neural Networks 1)

**Figure 1:**
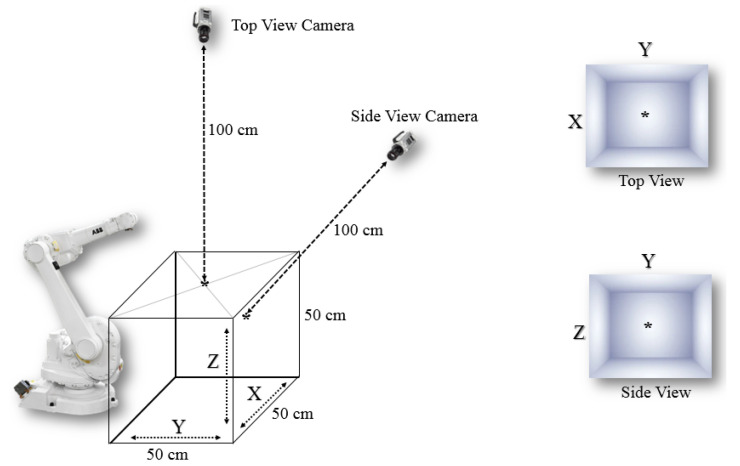
Workspace size and locations of cameras.

In the first stage, the coordinates of the bounding boxes of a start, target, and obstacle are obtained in the 3D space. These coordinates can be automatically determined for real-time applications. In the proposed approach, an object detection algorithm and a neural network were combined. In an object detection algorithm, both object recognition and object localization in an image can be achieved. These algorithms can distinguish between a start, an obstacle, and a target object, and extract their bounding box coordinates. Because we required 3D coordinates, we extracted XYZ coordinates using two cameras with two planes, as displayed in Figure 1.

Many object detection algorithms, including SqueezeDet, MobileNet, R-CNN, fast R-CNN, mask R-CNN, single-shot detector, and you only look once (YOLO), have been proposed [34,35,36,37,38]. An algorithm can be evaluated from several perspectives, such as speed of detection and accuracy of identification. Speed is particularly important because targets may constantly change, so target detection should be performed in real time, allowing new paths to be computed. In [39], the accuracy and speed of various object detection algorithms were compared. The results revealed that the YOLO algorithm is accurate and operates at a high speed. Therefore, the YOLO algorithm was chosen.

**Figure 2:**
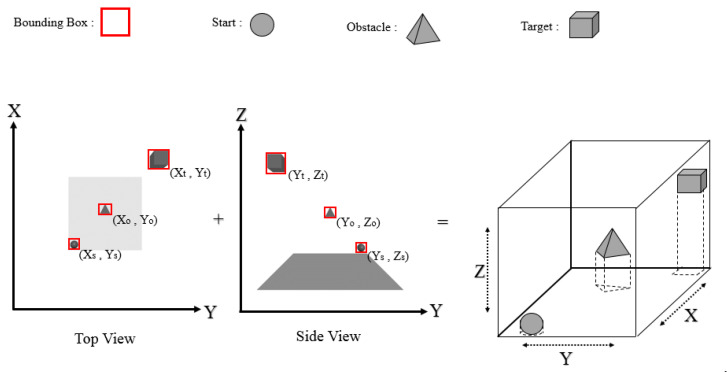
YOLO object detection in two views.

In YOLO, object detection is regarded as a regression problem involving spatially separated bounding boxes and associated class probabilities. Full images are evaluated once, and a single neural network predicts bounding boxes and class probabilities based on those images alone. The use of a single network to monitor the detection process allows the network to be tuned to obtain the best results. We mainly used one variant, tiny-YOLOv4, and extracted the bounding boxes of a start, an obstacle, and the target to find the approximate coordinates of the bounding boxes of all objects with perspective correction (Figure 2). The cells within the bounding box of two perpendicular planes were classified as objects; thus, the cells of objects in the 3D space were extracted.

We used three objects to represent a start, an obstacle, and a target in this study. A start, an obstacle, and a target object were represented by a sphere, a pyramid, and a cube, respectively, as displayed in Figure 2. A dataset was created based on these three classes, and photographs were labeled using the YOLO standard labeling method (where each photograph is labeled with LabelImg, which is a free, open-source tool for graphically labeling images). Table 1 presents the details of the dataset.

In this stage, the spatial coordinates of each detected object are determined. To this end, we used a trained neural network to determine the X-, Y-, and Z-coordinates of these three classes. The inputs of the neural network were the class of objects, the center, and the sizes of the bounding boxes obtained by the YOLO algorithm.

**Figure 3:**
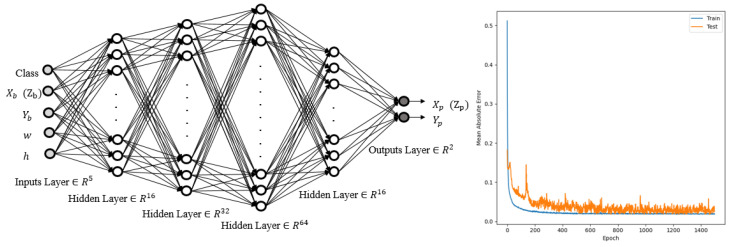
Structure of the first neural network and its mean absolute error convergence.

A six-layer neural network topology with four hidden layers containing 16, 32, 64, and 16 neurons was used. The outputs were the spatial coordinates of the corresponding plates. Figure 3 depicts the neural network architecture and the mean absolute error.

It is worth mentioning that we cannot use a simple mathematical problem to transform two 2D projected coordinates into a 3D coordinate. That is because many parameters influence the final accuracy of coordinates in real-world applications. Some of these factors are the focal length and distortion impact of the lens, camera sensor size, perspective effect, and positioning items behind another. When the focal length effect is paired with the perspective effect and the object is near the borders of an image, finding coordinates with high precision is a difficult operation using traditional mathematical approaches. Rather than a complex procedure, we proposed a combined YOLO-neural networks method to identify the coordinates of objects in pixel-based space with two cameras (YOLO) and convert the pixel-based space to 3D XYZ real-world coordinates (neural network). Using this method, we were able to extract 3D coordinates with acceptable precision in various positions of the objects. We could also reduce the detection time to roughly 0.04 s, allowing us to employ this approach in real-time applications.

**Figure 4:**
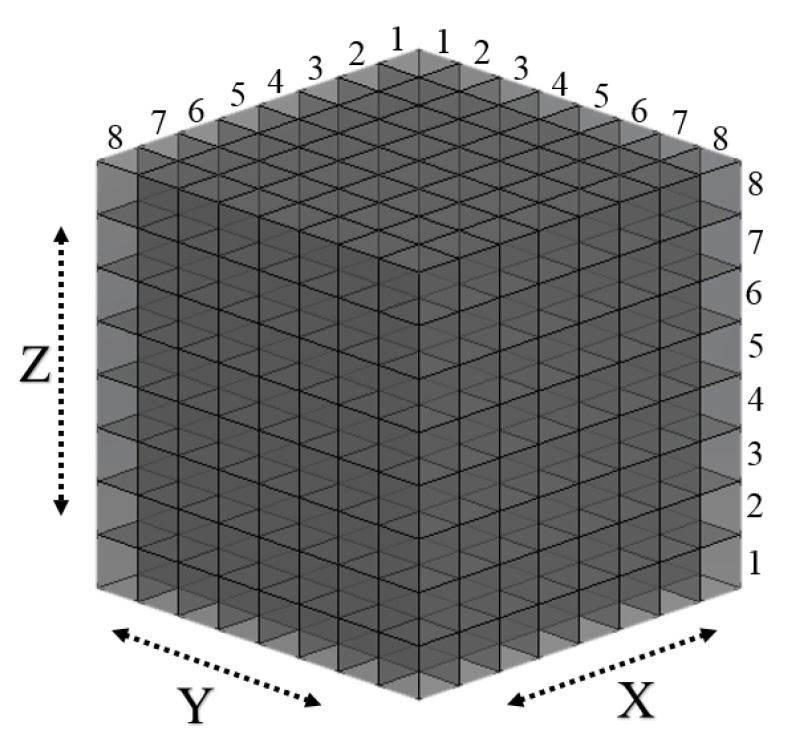
Gridded 3D workspace.

We transformed the dimensions of the 3D workspace from 50 × 50 × 50 cm to 8 × 8 × 8 discrete cells, as displayed in Figure 4. This conversion simplified and accelerated the overall calculation process. According to the grid workspace and the bounding boxes of the start, obstacle, and target, the corresponding start, obstacle, and target cells can be identified. These labeled cells are used in the Q-learning algorithm, as explained in the following section.

### 3.2. Action Finding (Q-Learning)

An action-finding process provides immediate action and state in the Q-learning technique of the cells after determining a start, an obstacle, and a target. In machine learning, Q-learning is an off-policy technique that focuses on how intelligent agents should function in a particular environment to optimize cumulative reward. An RL agent engages with its environment in discrete time steps. At each point *t*, the agent receives the current state $s_{\left\{t\right\}}$ and the reward $r_{\left\{t\right\}}$. As soon as action $a_{\left\{t\right\}}$ has been selected, it is conveyed to the environment. With each iteration, the environment changes to a new state ($s_{\left\{t+1\right\}}$), and the reward ($r_{\left\{t+1\right\}}$) associated with the change is computed (for each iteration). In RL, the projected total reward for the RL agent is maximized. This research utilized the Q-learning technique to identify a straightforward sequence of forward, backward, up, down, left, and right actions to maximize accumulated rewards. The method is inspired by the “windy grid world” problem. We divided the 3D workspace into several cells based on a designated resolution. As detailed in Sutton and Barto’s original “windy grid world” problem [40], the main purpose was to travel from one cell to its adjacent cells until the target cell was reached. The difference between this technique and the RL-based methods mentioned in the background section is that in this method, a direct action is determined to move from a cell (state) to its neighboring cells instead of finding a sequence of joint angles for the same movement. This assumption simplifies and speeds up the action-finding process. Using this approach, state space and action space can be expressed as follows:

(1) $$S=\left\{cell_{1},\;\;cell_{2},\;\;cell_{3},\;\;\ldots \;,\;\;cell_{n\;\times \;m\;\times \;l}\right\}.$$

The overall width, length, and height are represented by n, m, and l, respectively. The higher the required resolution is, the more cells will be needed. The total number of members in the state space is n×m×l.

(2) A={ Up,  Down,  Left,  Right,  Forward,  Backward}

In this study, the action space has six members, namely forward, backward, up, down, left, and right, as shown by Equation (2). The state space and the action space are small, and an algorithmic model can be easily developed for the interaction of a robot arm within this reduced environment. This technique can drastically reduce the execution time of an algorithm and simultaneously decrease its complexity.

Before commencing the Q-learning process to discover the optimum path, the locations of the start, target, and obstacle were identified. These three positions were randomly arranged in each test in the workspace. In this technique, the 3D workspace was a portion of the total workspace available in front of the robot arm.

We selected forward, backward, up, down, left, and right as the total action space. Although the number of actions can be increased, the computing cost increases. Additional improvements in terms of resolution, defined as the magnitude of the end-effector motion in each step, may exponentially increase the search space, which increases the computational burden. However, reducing the actions may lead to path discovery failure. Hence, there is a trade-off between speed and efficiency on the one hand and the probability of success on the other. The time required to run the Q-learning code is directly proportional to the number of cells. Therefore, establishing an appropriate resolution and speed for a specific application considering the dimensions of the robot arm and objects is critical. We used a 3D grid workspace of 8 × 8 × 8 cells (states) for this study, considering the sizes of the three objects and the speed of path planning. Therefore, this design implied a total of 512 cells (states) in which the end-effector could be placed. The start, target, and obstacle points were located within these cells. The Q-learning method was then used to determine the optimum actions from the start to the target cell under various reward and penalty scenarios.

Seijen revealed that in a “windy grid world” problem, Q-learning outperformed other algorithms, such as SARSA, to obtain the highest accumulated reward [41]. Therefore, we used Q-learning as the RL algorithm in the action-finding phase in this study. In this algorithm, if the robot arm successfully reaches the target cell, it earns a reward of 50 points. By contrast, if it reaches an obstacle cell, the agent receives a penalty of −100 points. Subsequently, all further acts result in a penalty of −1 point. The goal of the agent is simply to maximize its score. In the Q-learning algorithm, first, the best sequence of actions from the start cell to the target cell with avoidance of the obstacle cell is performed. A 3D grid workspace was set up as follows in this study:

- In an 8 × 8 × 8 3D grid workspace, six possible actions, namely forward, backward, up, down, left, and right, were considered.
- An agent starts from a randomly located start state and receives a reward of 50 points for reaching a randomly located target state.
- An agent receives a penalty of −100 points for reaching a randomly located obstacle cell.
- All other actions cause a penalty of −1 point.

This action-finding section created a 3D matrix of the robot arm’s most rewarding actions in each cell. These best actions can be followed from a start cell to a target cell to create the best actions or optimal path sequence. In the next section, we describe the conversion of these actions to angles that rotate the joints of a robot arm.

### 3.3. 6-DOF Angle Finding (Neural Networks 2)

In the final stage, we need to transform the sequence of actions into a sequence of joint angles. One way is the use of inverse kinematics (IK). The idea behind IK is to calculate the joint angles of the robot arm for a given position of the end-effector. The joint configurations needed for each intermediate time step along the trajectory are calculated. However, using IK in each step makes the method extremely slow. Another way is the use of a trained neural network. The neural network does not need to be trained every time; instead, it is trained only one time, and this trained neural network is used every time during path planning.

In this study, an angle-finding process is performed by using a trained neural network to obtain the joint angles of each specified action in a particular cell. We trained a neural network for angle finding using RoboDK software because RoboDK can provide a 3D model of our robot arm. Because the joint angles required to move the end-effector of a robot arm in each direction are dependent on the present state of the end-effector, the current cell must be considered by the neural network at each stage of the process. Therefore, the neural network must be fed with the indices of the row, column, and height of the cell as well as the action that has been selected as inputs. The outputs of the neural network should be the joint angles that supply the location of the next cell corresponding to the action. Notably, because the input must be in numerical form, actions such as up, down, left, right, forward, and backward are labeled numerically as 1, 2, 3, 4, 5, and 6, respectively. Furthermore, the number of outputs is proportional to the degrees of freedom (DOF) of the robot arm. The number of outputs in the robot arm in this research, with six joints, was therefore six.

We used a five-layer neural network topology with three hidden layers containing 4, 10, and 6 neurons. The number of hidden layers and neurons was determined empirically through trial and error during the training processes. Four inputs, namely the row, column, and height indices of a current cell, as well as an action to be performed, were considered. The robot arm used in this study, a model IRB 1600 with a 1.45 m arm length, had six DOF. The rectified linear unit (ReLU) is the activation function for input and output neurons because it is the most often implemented function and is not complex.

(3) $$\sigma_{ReLU}\left(x\right)=\max\left\{0,x\right\}$$

First, we collected datasets to train a neural network according to the designed structure by collecting precise data with an end-effector placed in various grid cells. We considered six neighboring cells of the central cell, where the end-effector was located, to be six data points. These six cells were those orthogonal to the central cell, and reachable by moving up, down, left, right, backward, or forward. We collected 3072 data points for neural network training. Of these data points, 90% were used in the learning process, and the rest were used in the testing and validation process. These data points were sufficient to train a neural network properly with an 8 × 8 × 8 grid workspace. Because the research used 512 cells (8 × 8 × 8 grid), 3072 moves were possible, and each operation in a cell could be performed in six directions. Thus, we used the entire set of information. Because the dataset was sufficiently large to include all cell movements, the dataset was considered adequate. Six possible outcomes were possible when the robot was in cells on the edge of the workspace. The robot ignored actions to move outside the workspace.

**Figure 5:**
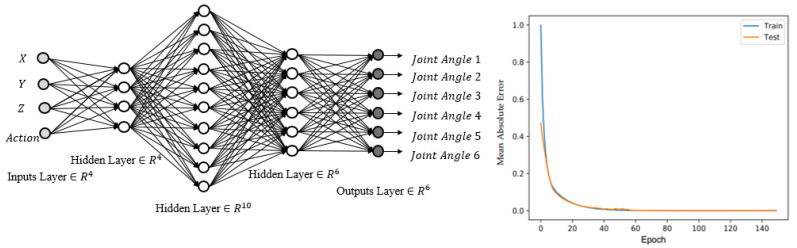
Structure of the second neural network and its mean absolute error convergence.

Initially, we trained a neural network with weights with random values. The output value was then calculated for each training sample. The weights were then updated using a backpropagation method and a gradient descent process. This procedure was repeated until the weights reached their optimal levels and the error ranges were within the permitted limits. Figure 5 depicts the neural network architecture and the mean absolute error.

A multilayer perceptron with a ReLU activation function first proposed by Paul Werbos [42] was used in this study. In this approach, a neural network is trained only once and then used for determining angles. A lengthy training period is thus reduced to a single step before path planning. The trained neural network required only a few seconds for use and could be used repeatedly.

The proposed computer vision-based path planning method can be summarized as follows:

1. Capturing a snapshot of the 3D workspace with the two cameras;
2. Detecting a start, target, and obstacle cell using the YOLO object-detection algorithm
3. Obtaining the spatial coordinates of three objects using the first neural networks;
4. Using the Q-learning method to determine an optimal route from a starting point to a target point while avoiding obstacle collision.
5. Finding the joint angles of the discovered actions using the trained neural network;
6. Implementation in the actual or simulated world of the acquired joint angle sequence on the robot arm.

**Figure 6:**
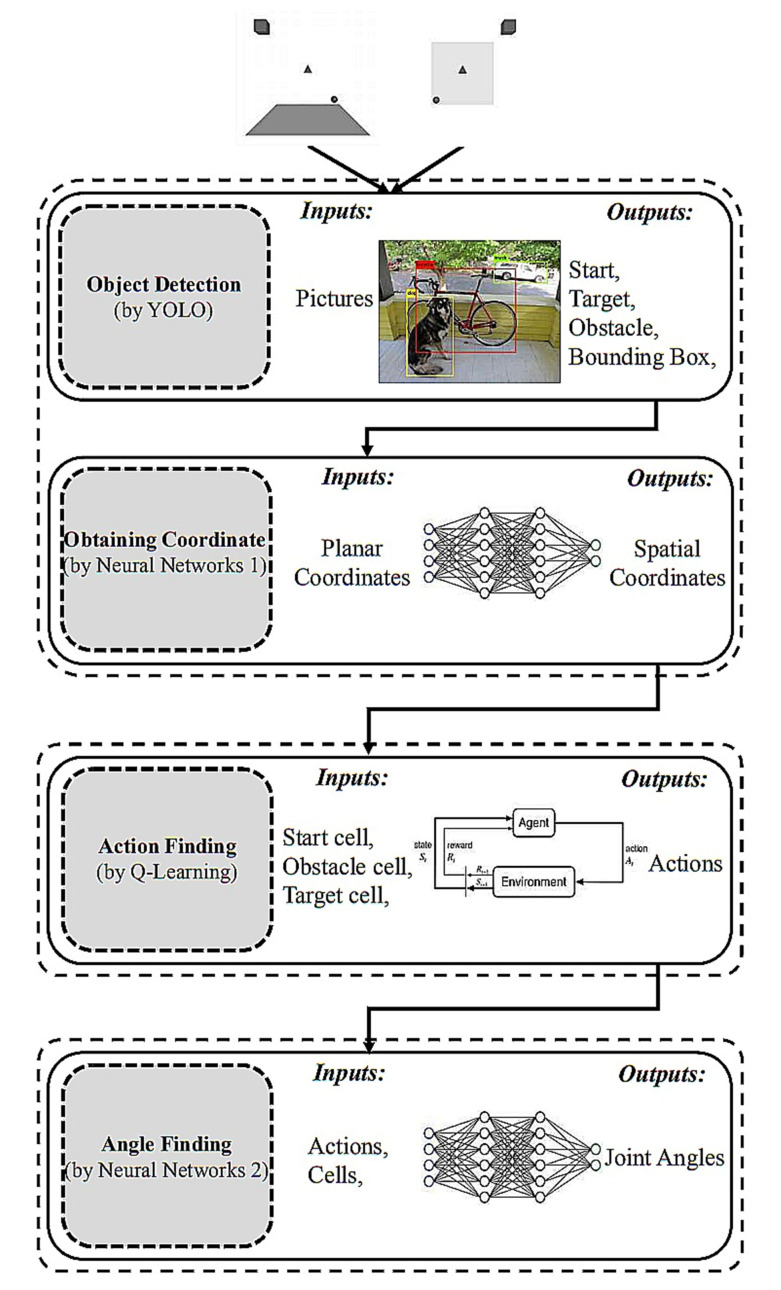
Method overview: 1. capturing pictures; 2. object detection by YOLO; 3. obtaining spatial coordinate by the first neural network; 4. action-finding by Q-learning; 5. angle-finding by the second neural network.

As displayed in Figure 6, we used a simple and fast technique in each phase of our process to provide an efficient path planning process. The use of low-quality images for the first stage could considerably speed up the picture analysis process because a start, an obstacle, and a target object shape could be identified from low-resolution photographs by using the algorithm. First, an object-detection method was used for object localization because it is one of the most accurate and rapid algorithms. Next, a trained neural network was used to obtain the spatial coordinates of these objects. We used the Q-learning algorithm to determine basic actions and determine the route with the highest reward. Next, the rapid conversion action of an experienced neural network was used for determining joint angles. In the next section, we discuss our testing of the effectiveness of the proposed method through simulation.

### 3.4. Simulation

To ensure that any proposed method is safe and provides desired results, simulation is essential before experimental implementation. First, we developed a 3D workspace by randomly locating a sphere, pyramid, and cube as a start, obstacle, and target object by using VPython, a library that allows users to create objects, such as spheres, cones, and other forms in 3D space. Two pictures displaying two views, namely the top and side views, were obtained. These two pictures are the inputs of the object-detection module. This module was configured to detect three classes of objects, namely a sphere, a pyramid, and a cube, in addition to finding the center of the bounding boxes as well as their dimensions.

For the next step, note that the outputs of the object detection (YOLO) algorithm were the inputs of the first neural network. Therefore, it has five inputs, namely (1) the class of an object, (2) the X- and (3) the Y- (or the Y- and the Z)-coordinates of the center of the bounding box, (4) the width, and (5) the length of the bounding box. It has two outputs, the X- and the Y- (or the Y and the Z) coordinates of an object in a spatial coordinate system. The coordinates of the center of the bounding box differ from the coordinates of the object in a spatial coordinate system because of the use of perspective pictures.

These spatial coordinates are then converted to a start, obstacle, and target cell. These cells are the inputs of the Q-learning algorithm, which finds the optimal path from the start cell to the target cell. In this simulation, we placed an obstacle object between the start and target objects to evaluate the performance of the Q-learning algorithm in terms of obstacle avoidance.

**Figure 7:**
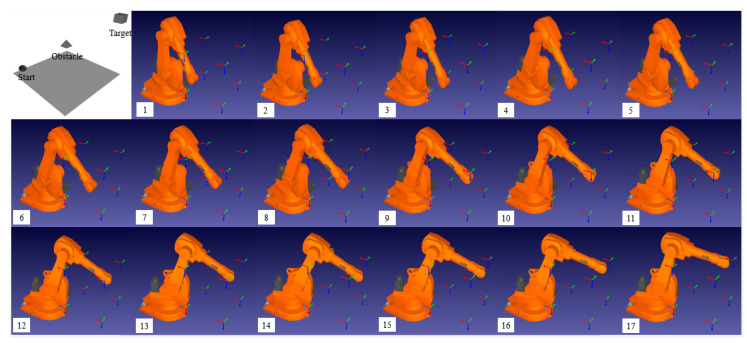
Simulation results in a RoboDK software: (**1**–**17**). The end-effector of a robot arm starts moving from a start point (Sphere) and avoid an obstacle point (Pyramid). Then reaches a target point (Cube).

Next, the outputs of the Q-learning algorithm, including a list of actions and cell indexes, were used as inputs for the second neural network. Thus, four inputs, including the X-, Y-, Z-coordinates of the current cell and its corresponding actions, existed in this neural network. The outputs were six joint angles that indicated the next cell of the optimal path. Next, the end-effector of a robot arm in a RoboDK simulator started moving from a start cell and followed the generated optimal path according to the obtained sequence of joint angles. Figure 7 displays the results of the simulation.

## 4. Experiment Results

**Figure 8:**
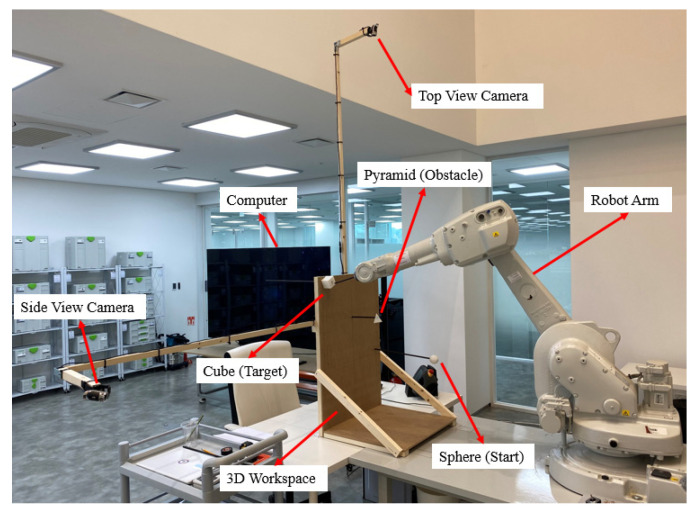
Experimental setup: cameras, robot arm, workspace, sphere (start), pyramid (obstacle), cube (target).

We tested the proposed method in a physical environment and verified its validity. The experimental setup (Figure 8) included a 6-DOF robot arm, two cameras, a computer, a 3D workspace, and three objects that represent a start, obstacle, and target point.

The test setup was similar to the simulated environment. The similarity was to enable comparison between the test results and simulation results. We installed two cameras and a robot arm, as was done in the simulation. Furthermore, a sphere (a start object), a pyramid (an obstacle object), and a cube (a target object) were placed in locations identical to those in the simulation. To ensure the physical test conditions were identical to those of the simulation, the specifications of the lens used in our cameras were identical to those of the virtual lens used in the simulation. Because of this similarity, the method presented in this article could be validated if physical robot test results were identical to the simulation result from the RoboDK software.

**Figure 9:**
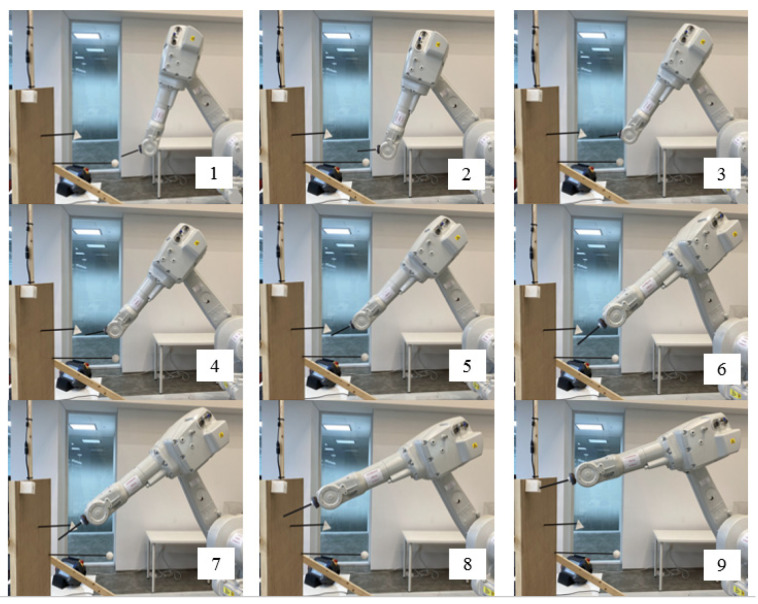
Real-world test: (**1**) The robot starts moving from the start point (Sphere). (**2**–**8**) The robot track the found path while avoiding the obstacle. (**9**) The robot reaches the target (Cube).

We placed a sphere (a start object) at the low-left corner, a pyramid (an obstacle object) in the middle, and a cube (a target object) at the upper-right corner of the 3D workspace. As displayed in Figure 9, the end-effector of the robot arm precisely followed the generated path, which was consistent with the results of the simulation test. Thus, the results of this physical test revealed that the proposed method exhibited satisfactory performance.

**Figure 10:**
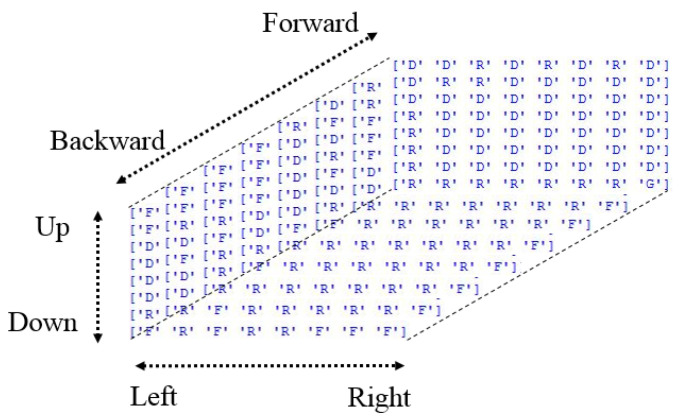
The optimal policy of each state (cell).

The results show that the robot follows the path well. This path was actually the best actions (optimal polices) of each cell (state) obtained by the Q-learning algorithm. When the Q-learning is running, it tries to find the best action that the agent can do in each state through interaction with the environment. Therefore, in each state, there is an action that is considered optimal action. In other words, the optimal policy is a 3D matrix (8 × 8 × 8) whose elements are a letter such as “U”, “D”, “L”, “R”, “B”, “F” that represent Up, Down, Left, Right, Backward, Forward respectively. Figure 10, displays the optimal policy of our test. The letter “G” and “O” (is not found in the Figure 10) represent Goal (or Target) and Obstacle cell.

What is very important is that the same optimal policy matrix can be used for each start point if the location of the target and obstacle remains fixed. The extracted path from the Q-learning results for our test is {F,F,F,F,R,F,F,F,D,R,R,D,D,D,D,D,D,R,R,R,R,G}. This sequence of optimal actions begins from the start cell in which the sphere is placed. If we change the location of the sphere, we do not require to run Q-learning again; we can use the same optimal policy matrix to find a new sequence of actions from the new start cell. This make the path generation more straightforward and fast.

Another important point is that in the Q-learning algorithm, there is an optimal episode limit. When the episode limit is too high, it takes a long time for an agent to become an experienced one. However, reducing the episode limit may lead to gaining not enough experience by the agent, which means it cannot find the best actions for states. Hence, there is a trade-off between speed on the one hand and gaining enough experience on the other. In this test, the optimal episode limit is equal to 2000, which guarantees speed and enough experience. This number was achieved through trial and error. In the next section, we discuss our method in general and give some suggestions for its improvement.

## 5. Discussion

In this section, we discuss the proposed method’s strengths and weaknesses. To this end, first, we compare our method with other methods in terms of speed which is a crucial feature in real-time path planning. To compare this method with other path planning methods, we refer to our previous study [9]. In that paper, we compared the speed of running time in our hybrid path planning method and other conventional path planning methods such as RL-based, APF, PRM, and RRT. In order to compare them, we ran a simple code of each method on the same computer to compute its running time. As we reported, our hybrid method required less running time to do the path planning process under the same conditions, which means it is faster than traditional methods due to using separate active and passive approaches. This comparison is discussed in detail in the previous work and can be referred to for more information.

In addition to strengths, this study has some limitations that should be taken into account in future studies. These limitations and our suggestions are presented in the following.

1. Although we tested the method using a physical robot arm, the physical setup was not exactly identical to the simulation because levitating multiple objects in the air and the middle of the workspace is difficult due to the use of fixtures and wood structures. Certain parts of a robot arm, such as the end-effector, may collide with fixtures. Therefore, in the future, the feasibility of using augmented reality (AR) for virtual test setup in physical robot tests should be considered. Furthermore, the efficacy of using a virtual sphere, pyramid, and cube instead of using physical objects that require fixtures should be evaluated. Such augmented tests can considerably reduce expenses and facilitate the rapid implementation of complex settings. Moreover, a more comprehensive analysis is possible than in an experimental test.
2. In future studies, trained deep Q-learning can also be incorporated into the proposed method to learn additional possible pathfinding to increase the speed of the action finding section. To use a deep Q-learning technique, a large dataset is required, which is currently not possible. This approach can drastically reduce the inference time, but concerns regarding high computing costs remain.
3. We used two cameras in two views which made our method less practical, especially for those applications that need a portable robot arm. One suggestion is that we use a single perspective picture taken by deep cameras then generate a path.
4. Our invention just takes shots at the beginning and a robot moves following a generated path. In order to be a realistic application, it should take many pictures (one per sec) and re-generate a path. Therefore, the system could be an intelligent real-time system.
5. We used a combined object detection-neural network method to calculate its spatial coordinates. It is suggested using 3D reconstruction methods to calculate objects’ position.
6. To make the method more practical, further study may use actual objects such as a cup, pen, monitor, book, etc.

In the end, in order to make the proposed method more useful and practical, a list of useful applications is mentioned.

- It could be used for a harvesting robot to collect fruits from trees avoid obstacles.
- It could be used for a recycling robot to pick up bottles, cans, batteries, or other particular objects in a recycling factory in real-time.
- It could be used for warehouse robots to pick selected items from shelves or totes and place them into shipping containers to fulfill orders.
- It could be used for assembly line robots to pick components and place them at an appropriate location.

## 6. Conclusions

A novel computer vision approach was proposed for effective path planning by combining Q-learning and neural networks for robot arms. In the proposed approach, computer vision and neural networks were combined to obtain accurate spatial locations of a start, an obstacle, and a target object in real time. In the 3D workspace, a sphere, a pyramid, and a cube were used to represent the start, obstacle, and target points, respectively. Two images from two views were inputted into a trained YOLO algorithm to detect the aforementioned items and find bounding boxes for each detected object. A trained neural network converted the bounding boxes into spatial coordinates. Next, a Q-learning algorithm determined the optimal sequence of actions in a 3D workspace from a start cell to a target cell and simultaneously avoided obstacles. Next, a trained neural network converted the identified actions to the corresponding joint angles. Because the neural networks were trained before the path planning process, the method was fast. We tested this computer vision-based path planning algorithm through simulation and experimental methods. The results revealed that this research overcame the limitations of our previous research [9].

Future research directions are suggested as follows. First, the feasibility of using augmented reality (AR) for virtual test setup in physical robot tests could be considered. Second, the use of a trained deep Q-learning can also be considered to increase the speed of the action finding section. Third, the use of a single perspective picture taken by deep cameras could be considered to avoid using two cameras. Forth, instead of using pictures only one time, a live video can be used to make the method more real-time. Fifth, the use of 3D reconstruction methods to calculate objects’ positions could be considered. Sixth, the use of actual objects such as a cup, pen, monitor, book, etc. could be considered to make the method more practical.

## Figures and Tables

**Table 1:** Details of the dataset.

Shape	Number of Data	Class
Pyramid	200	0
Sphere	200	1
Cube	200	2

## Data Availability

Not applicable.

## Abbreviation

The following abbreviations are used in this manuscript:

APF	artificial potential field
PRM	probabilistic road maps
RRT	rapidly-exploring random tree
RL	reinforcement learning
DOF	degrees of freedom
